# Initial Development of the DIGNITY Program: Balancing Autonomy and Safety in Dementia Care

**DOI:** 10.1093/geroni/igaf122.2067

**Published:** 2025-12-31

**Authors:** Liza Behrens, Kimberly Van Haitsma, Jennifer Kraschnewski, Marie Boltz

**Affiliations:** The Pennsylvania State University, University Park, Pennsylvania, United States; The Pennsylvania State University, University Park, Pennsylvania, United States; Penn State College of Medicine, Hershey, Pennsylvania, United States; The Pennsylvania State University, University Park, Pennsylvania, United States

## Abstract

In the name of safety, nursing Home (NH) residents living with dementia often have their preferences for care and daily activities disregarded by NH staff. This impinges on residents’ rights to exercise autonomy in clinical decision-making and leads to dissatisfaction with care comprising quality of life and well-being. No comprehensive evidence-based risk management strategies exist to assist NH staff to identify and support care choices of residents while attempting to balance autonomy with safety. Thus, the purpose of this project was to use community engagement intervention mapping techniques to develop a multilevel intervention designed to support shared decision-making with NH residents living with dementia thus promoting resident health and autonomy. This presentation will describe the early planning phase of intervention mapping including the construction of a logic model of the problem, a review of literature identifying behavioral, normative, and control beliefs associated with NH staff intentions to honor residents’ preferences, and a set of training goals and objectives. This project resulted in a multi-component intervention that guides shared decision-making in NHs to support person-centered dementia care called DIGNITY (Decision-making in aging and dementia for autonomy). It includes an adapted care planning protocol and staff training to help direct-care NH staff negotiate intrinsic and cultural factors in preference situations that carry a risk to residents’ health and safety. Initial development of this intervention sets the stage for initial program manual and tool development necessary for pilot testing the intervention in the future.

